# Effects of Six Months Training on Physical Capacity and Metaboreflex Activity in Patients with Multiple Sclerosis

**DOI:** 10.3389/fphys.2016.00531

**Published:** 2016-11-14

**Authors:** Sara Magnani, Sergio Olla, Massimiliano Pau, Girolamo Palazzolo, Filippo Tocco, Azzurra Doneddu, Maura Marcelli, Andrea Loi, Federica Corona, Francesco Corona, Giancarlo Coghe, Maria G. Marrosu, Alberto Concu, Eleonora Cocco, Elisabetta Marongiu, Antonio Crisafulli

**Affiliations:** ^1^Sports Physiology Lab, Department of Medical Sciences, University of CagliariCagliari, Italy; ^2^Department of Mechanical, Chemical and Materials Engineering, University of CagliariCagliari, Italy; ^3^Department of Public Health, Clinical and Molecular Medicine, University of CagliariCagliari, Italy

**Keywords:** cardiovascular regulation, systemic vascular resistance, blood pressure, stroke volume, cardiac output

## Abstract

Patients with multiple sclerosis (MS) have an increased systemic vascular resistance (SVR) response during the metaboreflex. It has been hypothesized that this is the consequence of a sedentary lifestyle secondary to MS. The purpose of this study was to discover whether a 6-month training program could reverse this hemodynamic dysregulation. Patients were randomly assigned to one of the following two groups: the intervention group (MSIT, *n* = 11), who followed an adapted training program; and the control group (MSCTL, *n* = 10), who continued with their sedentary lifestyle. Cardiovascular response during the metaboreflex was evaluated using the post-exercise muscle ischemia (PEMI) method and during a control exercise recovery (CER) test. The difference in hemodynamic variables such as stroke volume (SV), cardiac output (CO), and SVR between the PEMI and the CER tests was calculated to assess the metaboreflex response. Moreover, physical capacity was measured during a cardiopulmonary test till exhaustion. All tests were repeated after 3 and 6 months (T3 and T6, respectively) from the beginning of the study. The main result was that the MSIT group substantially improved parameters related to physical capacity (+5.31 ± 5.12 ml·min^−1^/kg in maximal oxygen uptake at T6) in comparison with the MSCTL group (−0.97 ± 4.89 ml·min^−1^/kg at T6; group effect: *p* = 0.0004). However, none of the hemodynamic variables changed in response to the metaboreflex activation. It was concluded that a 6-month period of adapted physical training was unable to reverse the hemodynamic dys-regulation in response to metaboreflex activation in these patients.

## Introduction

Exercise training is a well-established intervention to improve physical capacity in many types of diseases. Beneficial effects have been reported in heart failure, chronic ischemic heart disease, hypertension, diabetes, and other metabolic conditions. For example, training has been found capable to improve physical capacity and quality of life in heart failure, to reduce blood pressure, to increase survival after infarction, and to increase insulin sensitivity (Pedersen and Saltin, 2015). Recently, improvements in symptoms and quality of life have also been demonstrated in neurological pathologies. Among others, Multiple Sclerosis (MS) has been found to be positively affected by physical exercise, as peak oxygen uptake and oxygen pulse were found improved after periods of training, thereby suggesting an increase exercise tolerance in these patients. Moreover, training may induce a positive effect on MS fatigue (Rampello et al., 2007; Dalgas et al., 2008; Andreasen et al., 2011; Pedersen and Saltin, 2015). However, data on the effect of supervised physical training on hemodynamic regulation during exercise in MS patients are scarce.

One of the key symptoms in MS is exertion fatigue, which is reported by 50–60% of these patients as their most disturbing problem (Tartaglia et al., 2005; Mollaouğlu and Űstűn, 2009; Andreasen et al., 2011). Several reasons might be responsible for this phenomenon. Of these, cardiovascular dysregulation during exercise has been observed in some investigations dealing with MS (Ng et al., 2000; Sanya et al., 2005). Some individuals with MS may have impairment of the autonomically-mediated blood pressure response during isometric exercise, and this phenomenon has been suggested to be related to symptomatic fatigue (Ng et al., 2000).

In healthy subjects, hemodynamic regulation during exercise is achieved by the integration of signals arising from mechano- and metabo-receptors within the working muscle and central command activation, which together concur in increasing sympathetic activity (O'Leary et al., 2004; Amann et al., 2011; Nobrega et al., 2014). The increased sympathetic activity is modulated in turn by the baroreflex activation, which dampens any excessive increment in sympathetic tone (Crisafulli et al., 2015). Several clues suggest that MS can influence these cardiovascular reflexes, thereby impairing the hemodynamic response during exertion (Ng et al., 2000; Flackenecher et al., 2001; Sanya et al., 2005; Marongiu et al., 2013). For instance, it was found impaired baroreflex control of the HR rate in MS patients, as well as impairment of the sympathetic-mediated baroreflex control of blood vessels, which together can explain the high incidence of orthostatic dizziness in these patients (Sanya et al., 2005). Moreover, our group has recently demonstrated that MS patients had an altered hemodynamic response to metaboreflex activation (Marongiu et al., 2015). This response resembled that showed by patients with cardiovascular and metabolic disease, where the target blood pressure is reached mainly by means of vasoconstriction (Piepoli et al., 2008; Delaney et al., 2010; Dipla et al., 2010; Choi et al., 2013; Crisafulli et al., 2013; Milia et al., 2015b). In healthy subjects, during the metaboreflex, a cardiac output-mediated mechanism is primarily responsible for the normal increase in mean blood pressure (MBP; Crisafulli et al., 2003, 2008; Delaney et al., 2010; Roberto et al., 2012; Spranger et al., 2013). In the investigation by Marongiu et al. (2015), it was hypothesized that physical deconditioning was responsible for the exaggerated vasoconstriction and the increase in SVR found in MS during the metaboreflex. This hypothesis is supported by previous findings suggesting that deconditioning may play a key role in the impaired exercise tolerance of people with MS (Rampello et al., 2007). Moreover, others have reported a shift in fiber type composition from type I fibers to a greater proportion of type II fibers in MS patients as a consequence of deconditioning (Dalgas et al., 2008). This occurrence may potentially have an impact on the metaboreflex activation as metabolites production is related to fiber type, with type II producing higher amounts of end-products than type I. It is then reasonable to assume that a shift from type I to type II in the muscle fiber composition would result in a more robust metaboreflex activity.

Therefore, we wondered whether a physical training program, along with its effects on physical capacity, could also counteract the reported cardiovascular dysregulation to the metaboreflex in these patients. To address this question, two groups of patients with MS were studied: one underwent a program of adapted physical activity (APA) and the other did not. The cardiovascular response to the muscle metaboreflex was then assessed at baseline, at three, at 6 months from the beginning of the study. To detect whether or not improvements in the capacity to exercise were paralleled by improvements in the hemodynamic response to the metaboreflex activation, indices of physical capacity were also evaluated.

## Materials and methods

### Study population

Twenty-five MS patients were selected from the database of the Multiple Sclerosis center of the Department of Public Health, Clinical and Molecular Medicine, of the University of Cagliari, Italy. Inclusion criteria were: (1) clinically established diagnosis of MS, defined according to the criteria proposed by Poser et al. (1983); (2) age between 18 and 65 years; (3) no relapse or deterioration during the 4 weeks prior to tests; (4) absence of other associated medical conditions that would interfere with the autonomic function and/or chronic cardiopulmonary diseases; (5) absence of involvement in any training program. Patients taking β-blockers, sympatho mimetics, anticholinergics, Amantadin, and/or tricyclic antidepressants were excluded. Complete neurological and medical history were obtained from each patient. Neurological examination was conducted and disability was evaluated according to the Expanded Disability Status scale (EDSS), which yielded an average value of 3.5 ± 0.7 (range 1.4–4.5; Kurtkze, 1983). At the time of the study, patients were on treatment with disease modifying drugs mainly, interferon-β, glatiramer acetate, fingolimod, or natalizumab.

A computer program was used to randomly assign patients to one of the following two groups:

Intervention MS group [MSIT group; a cohort of 13 patients (6 women and 7 men)]. The MSIT underwent a 6-month supervised training program (STP) including both aerobic and strength training. The 6-month period was chosen because it was reported that in MS patients substantial improvement in physical capacity can be obtained with short periods (2–6 months) of supervised physical activity (Rampello et al., 2007; Dalgas et al., 2008). Briefly, patients participated in three training sessions per week. Each session lasted 1 h and consisted in a 10-min warm-up on an electromagnetically braked cycle ergometer (Forma Bike 5000, Technogym, Forlì, Italy) at 30% of the maximum workload previously calculated during a cardiopulmonary test (CPT). Patients then performed stretching exercises of their upper and lower limbs and trunk muscles. This was followed by 20 min of aerobic training obtained by cycling at a work rate corresponding to 50% of their maximum. This workload was progressively increased every week up to 80% of maximum work rate, and adjusted every 3 months, taking into consideration results obtained from the CPT, which was repeated every 3 months. After this period of aerobic training, patients were instructed to perform gait exercises, such as ambulation combining advancement of one lower limb with raising of the opposite upper limb and tandem gait. Then, the period of strength training began and involved muscles of the upper and lower limb and trunk. In terms of sets, repetitions and load, patients started with 1 set of 8 repetitions for each muscle group at a load corresponding to 15% of the maximum. Load and sets were progressively increased to three sets of 12 repetitions at a load corresponding to 30% of the maximum. Between sets, a rest period of ~2–3 min was allowed. At the end of each training session, a 5-min cool-down was further allowed. Adherence to the protocol was considered satisfactory if patients attended at least 80% of the total training sessions.

Control MS group (MSCTL group), comprising 12 MS patients (5 women and 7 men). The MSCT group patients were not involved in any training program and continued their normal lifestyle.

The study protocol conforms to the declaration of Helsinki and was approved by the local ethical committee (Comitato Etico dell'azienda Ospedaliero-Universitaria di Cagliari, approval number 180 of the October 17th, 2012). Written informed consent was obtained from all participants.

### Experimental design

Each patient was followed for 6 months and reported to our Lab 3 times: at the beginning of the study (T0), and at the 3rd and 6th month after T0 (T3 and T6, respectively). On each occasion, they underwent the following study protocol: a general medical examination with ECG followed by an incremental exercise on an electro-magnetically-braked cycle ergometer (CUSTO Med, Ottobrunn, Germany) to assess their physical capacity. The test consisted of a linear increase of workload (10 W/min), starting at 10 W, at a pedaling frequency of 60 rpm, until exhaustion which was taken as the point at which the subject experienced muscle fatigue (i.e., was unable to maintain a pedaling rate of at least 50 rpm). During the incremental test, subjects were connected to a gas analyzer (Ultima CPX, MedGraphics, St. Paul, MN) to perform a CPT. Values of oxygen uptake (VO_2_), carbon dioxide production (VCO_2_), pulmonary ventilation (VE), and heart rate (HR) were collected. Afterwards, data from the CPT were analyzed to calculate individual anaerobic threshold (AT), which was detected by using the V slope method. Briefly, a computerized regression analysis of VO_2_ slopes vs. VCO_2_ plot during exercise was employed (Beaver et al., 1986). Values VO_2_, VE, HR, and workload (W) at AT were gathered (VO_2AT_, VE_AT_, HR_AT_, W_AT_). Maximum oxygen uptake (VO_2max_) was calculated as the average VO_2_ during the final 30 s of the exercise test. Achievement of VO_2max_ was considered as the attainment of at least two of the following criteria: (1) a plateau in VO_2_ despite increasing workload (<80 mL min^−1^); (2) respiratory exchange ratio (RER) above 1.10; and 3) heart rate ± 10 beats·min^−1^ of predicted maximum heart rate (HR) calculated as 220-age (Howley et al., 1995). Moreover, maximum VE (VE_max_), HR (HR_max_), and workload (W_max_) were calculated.

After the CPT session (the interval was at least 3 days, range 3–7 days), each subject was randomly assigned to the following protocol to study metaboreflex activity:

- post-exercise muscle ischemia session (PEMI session), which encompassed 3 min of resting, followed by 3 min of exercise consisting in rhythmic dynamic handgrip at 30 compressions/min at 30% of the maximum assessed as the peak reached during five previous maximal compressions on a hydraulic dynamometer (MAP 1.1, Kern, Balingen, Germany). Exercise was followed by PEMI on the exercised arm. PEMI was induced by rapidly (in <3 s) inflating an upper arm biceps tourniquet to 50 mmHg above peak exercise systolic pressure. The ischemia lasted 3 min. Three minutes of recovery were further allowed after the cuff was deflated, for a total of 6 min of recovery. This protocol has been shown to trap the muscle metabolites in the exercising limb and to maintain stimulation of the metaboreceptors (Crisafulli et al., 2007, 2011; Marongiu et al., 2013). This protocol has been demonstrated to induce substantial cardiovascular activation in terms of enhancement in stroke volume (SV), SVR, cardiac contractility, and cardiac pre-load (Crisafulli et al., 2007, 2008, 2011; Roberto et al., 2012; Milia et al., 2015a).
- control exercise recovery session (CER session): the same rest-exercise protocol used for PEMI was performed followed by a control exercise recovery of 6 min without tourniquet inflation. Sessions A and B were spaced by at least 1 day (interval 1–5 days) and were spaced by the last exercise session by at least 24 h.

All experiments were carried out in a temperature-controlled, air-conditioned room (temperature set at 22°C and relative humidity 50%). All experiments were conducted in the morning (between 10.00 and 12.00 A.M.). Patients were instructed to have breakfast at least 2 h before coming to our laboratory and to avoid caffeine and alcohol ingestion the day before sessions were scheduled.

During both the PEMI and the CER test, hemodynamics was assessed by means of impedance cardiography (NCCOM 3, BoMed Inc., Irvine, CA), which has already been used in similar experimental settings (Crisafulli et al., 2007, 2008, 2013; Roberto et al., 2012). The data acquisition method is described in detail in our previous published papers (Crisafulli et al., 2007, 2008, 2013; Roberto et al., 2012). Briefly, by using a digital chart recorder (ADInstruments, PowerLab 8sp, Castle Hill, Australia) NCCOM 3-derived analog traces of ECG, thorax impedance (Z0), and Z0 first derivative were recorded and stored. After, the Sramek-Bernstein equation (Bernstein, 1986) was applied to calculate beat-to-beat SV from stored trans-thoracic impedance traces. The pre-ejection period/left ejection time ratio (PEP/VET) was also calculated from impedance traces, as shown in previous papers (Crisafulli et al., 2003, 2007, 2008). This ratio shows a good correlation with the angiographic ejection fraction and represents an inverse index of myocardial performance (Lewis et al., 1977). Diastolic time (DT) was measured by subtracting the sum of PEP and VET from the cardiac cycle total period and, by dividing SV by DT, the ventricular filling rate (VFR) was obtained (Marongiu et al., 2013). VFR is a measure of the mean rate of diastolic blood flux. HR was considered as the reciprocal of the electrocardiogram R-R interval and cardiac output (CO) was obtained by multiplying SV•HR. Subjects were also connected to a manual sphygmomanometer for systolic (SBP) and diastolic (DBP) blood pressure assessment, which was measured in the non-exercised arm by the same physician throughout all protocol sessions. To calculate MBP, the formula previously described by Moran et al. (1995) which assesses MBP by taking into account changes in the diastolic and systolic periods was used. SVR was obtained by multiplying the MBP/CO ratio by 80, where 80 is a conversion factor to change units to standard resistance units.

### Data analysis and calculation

Descriptive statistics were performed on each variable to confirm the assumptions of normality by means of the Kolmogorov-Smirnov test. The alpha level was set at *p* < 0.05. Differences between groups in means ± standard deviation (SD) of anthropometric characteristics, CPT and hemodynamic variables at T0 were carried out by unpaired *t*-test. Differences between groups in means ± *SD* of variation from T0 in CPT parameters reached by subjects at T3 and T6 were assessed by means of two-way analysis of variance (ANOVA) for repeated measures for the effects of group and time followed by Tukey *post-hoc* when appropriate.

Hemodynamic responses during PEMI and CER tests were averaged over 1 min. For each variable, the values at the third minute of recovery from the tests (when a steady state was expected to be reached) were taken into account. To assess metaboreflex activity, the following procedure was employed: for each parameter, the difference between the PEMI and the CER test was calculated. This procedure allowed us to assess metaboreflex response, i.e., the response due to the metaboreflex activity (Milia et al., 2015a,b; Crisafulli et al., 2013). Differences between groups in mean ± *SD* of measured variables due to metaboreflex response were assessed by means of two way ANOVA for repeated measures (factors: group and time) followed by Tukey *post-hoc* when appropriate. Statistical analysis was carried out utilizing commercially available software (GraphPad Prism, La Jolla, CA). Statistical significance was established as a *p* < 0.05 in all cases.

## Results

Four of the 25 patients (two from the MSIT and two from the MSCTL group, one male and one female for each group) abandoned the study. In detail, three of them had a clinical deterioration which caused a substantial increment in the EDSS scale, while one had a car accident which caused immobilization for 3 months. Thus, data shown are from 11 MSIT patients and 10 MSCTL patients. Their physical characteristics, respectively, for MSIT and MSCTL were: age 47.8 ± 10.8 and 40.7 ± 14 years (*p* = 0.20), body mass 68.5 ± 17.6 kg and 64.5 ± 10.1 kg (*p* = 0.50), height 168.4 ± 8 cm and 166 ± 9.2 cm (*p* = 0.53). There was no difference in any parameters between groups. Table 1 shows data from the CPT-test collected at T0. Data of VO_2_ are expressed indexed by body weight. No statistical differences were observed between groups. Table 2 reports mean values of hemodynamic parameters recorded during rest periods preceding the PEMI and the CER tests at T0, T3, and T6, respectively. Statistics did not evidence any difference due to group or condition. Table 3 shows hemodynamic data collected at the third minute of recovery of the PEMI and the (CER) tests. Responses in parameters are also shown.

**Table 1:** **Data from the cardiopulmonary test gathered at T0 in the control Multiple Sclerosis group (MSCTL, ***n*** = 10) and in the intervention Multiple Sclerosis group (MSIT, ***n*** = 11) group**.

	**MSCTL**	**MSIT**	***p*-value**
VO_2AT_ (mL/kg·min^−1^)	14.1±5.6	13.3±4.3	0.71
VE_AT_(L·min-1)	28.1±10.5	25.4±8.2	0.44
HR_AT_(bpm)	111.4±25.2	111.4±11.11	0.99
W_AT_	60±24.9	57.3±23.7	0.79
VO_2max_(mL/kg·min^−1^)	22.2±7.6	18.6±7.7	0.29
VE_max_(L·min^−1^)	52.9±2	49.5±31.3	0.76
HR_max_ (bpm)	136.8±24.1	136.5±17.4	0.97
W_max_	99±31.8	92.7±36.6	0.68

*Parameters were collected at anaerobic threshold and at maximum exercise. Values are mean ± SD*.

**Table 2:** **Hemodynamic data values during rest periods preceding post-exercise muscle ischemia (PEMI) and control exercise recovery (CER) tests at T0, T3, and T6 in both control Multiple Sclerosis group (MSCTL, ***n*** = 10) and in the intervention Multiple Sclerosis group (MSIT, ***n*** = 11) group**.

	**MSCTL (T0)**	**MSIT (T0)**	***p*-value (group and condition effect)**	**MSCTL (T3)**	**MSIT (T3)**	***p*-value (group and condition effect)**	**MSCTL (T6)**	**MSIT (T6)**	***p*-value (group and condition effect)**
**HR (bpm)**									
Rest before PEMI	71.1±12.7	72.4±8.1	0.94	70.0±10.1	70.4±11.5	0.78	76.9±14.8	73.3±9.5	0.43
Rest before CER	72.7±12.7	71.8±9.3	0.88	69.2±8.8	70.6±11.6	0.92	73.1±12.8	70.7±12.3	0.40
**SV (ml)**									
Rest before PEMI	68.8±12.1	59.5±16.7	0.17	61.3±17.6	62.2±19.3	0.81	57.8±16.6	60.0±16.6	0.80
Rest before CER	63.4±15.7	59.9±14.6	0.57	59.2±15.8	61.0±21.2	0.78	60.8±15.2	61.3±21.4	0.69
**CO (l**·**min**^−1^**)**									
Rest before PEMI	4.9±0.9	4.3±1.3	0.35	4.2±0.9	4.2±0.9	0.78	4.3±1.2	4.4±0.9	0.65
Rest before CER	4.4±1.2	4.3±1.4	0.53	4.2±1.0	4.1±0.9	0.67	4.3±1.1	4.5±1.1	0.88
**PEP/VET**									
Rest before PEMI	0.5±0.2	0.6±0.1	0.78	0.52±0.1	0.50±0.1	0.29	0.53±0.1	0.57±0.2	0.65
Rest before CER	0.6±0.2	0.6±0.1	0.80	0.55±0.1	0.51±0.1	0.64	0.55±0.2	0.56±0.2	0.92
**MBP (mmHg)**									
Rest before PEMI	92.2±12.9	89.4±11	0.63	89.5±11.3	93.4±9.2	0.10	85.1±12.3	87.7±10.4	0.18
Rest before CER	92.6±14.8	92±9	0.68	88.4±13.5	95.1±7.2	0.92	84.6±10.4	91.6±12.9	0.63
**SVR (dyne**·**s**·**cm**^−5^**)**									
Rest before PEMI	1545.9±418.5	1863.1±838.8	0.33	1827.0±637.8	1864.3±540.8	0.96	1686.9±553.8	1445.2±242.5	0.13
Rest before CER	1789±661	1891.6±776.7	0.53	1878.9±691.5	1859.7±488.3	0.90	1693.0±675.2	1494.0±307.3	0.85
**VFR (ml**·**s**^−1^**)**									
Rest before PEMI	164.1±54	140.9±52.5	0.36	122.3±29.9	115.5±28.4	0.54	133.6±51.7	154.6±36.6	0.12
Rest before CER	151.2±70.4	140.8±58.9	0.72	117.5±27.2	113.8±26.2	0.70	131.7±48.7	154.9±42.7	0.95

*HR, heart rate; SV, stroke volume; CO, cardiac output; PEP/VET, pre-ejection period/ventricular ejection time ratio; MBP, mean blood pressure; SVR, systemic vascular resistance; VFR, ventricular filling rate. Values are mean ± SD*.

**Table 3:** **Hemodynamic data at the third minute of recovery of post-exercise muscle ischemia (PEMI) and control exercise recovery (CER) tests at T0, T3, and T6 in both control Multiple Sclerosis group (MSCTL, ***n*** = 10) and in the intervention Multiple Sclerosis group (MSIT, ***n*** = 11) group**.

	**MSCTL (T0)**	**MSIT (T0)**	**MSCTL (T3)**	**MSIT (T3)**	**MSCTL (T6)**	**MSIT (T6)**
HR (bpm)	72.2±13.2	73.4±10.6	69.5±10.7	70.4±11.0	72.4±12.3	73.1±10.4
PEMI	71.6±11.5	71.9±9.5	69.2±9.7	72.7±13.4	71.5±11.4	69.9±11.5
CER	Δ = 0.5±3.8	Δ = 1.4±2.6	Δ = 0.2±3.6	Δ = 2.2±6.0	Δ = 0.9±5.1	Δ = 3.1±5.3
SV (ml)	67.4±16.7	57.1±16.0	60.9±15.5	57.5±15.9	61.2±13.2	73.1±15.1
PEMI	67.4±20.3	58.8±14.5	60.5±15.6	60.0±15.1	60.5±14.5	71.4±14.7
CER	Δ = 0.01±6.3	Δ = −1.6±3.2	Δ = 0.4±4.1	Δ = −2.4±4.2	Δ = 0.5±4.7	Δ = 0.6±2.3
CO (l·min-1)	4.8±1.3	4.1±1.4	4.2±1.1	4.1±1.0	4.4±1.1	5.1±1.2
PEMI	4.7±1.5	4.2±1.2	4.1±0.9	4.2±0.7	4.3±0.9	4.9±1.0
CER	Δ = 0.1±0.6	Δ = 0.03±0.5	Δ = 0.04±0.5	Δ = −0.02±0.7	Δ = 0.07±0.4	Δ = 2.4±0.4
PEP/VET	0.5±0.1	0.5±0.1	0.5±0.1	0.4±0.1	0.4±0.1	0.4±0.1
PEMI	0.5±0.1	0.5±0.1	0.5±0.09	0.4±0.09	0.4±0.09	0.4±0.09
CER	Δ = −0.03±0.04	Δ = 0.008±0.06	Δ = 0.01±0.05	Δ = −0.01±0.04	Δ = −0.01±0.05	Δ = 0.01±0.06
MBP (mmHg)	100.7±16.9	100.0±9.2	96.2±6.7	97.2±11.4	92.8±7.1	94.5±6.3
PEMI	95.6±16.7	89.6±9.6	89.4±6.7	91.8±9.2	85.0±5.2	90.3±5.5
CER	Δ = 5.1±6.0	Δ = 10.3±6.3	Δ = 6.7±6.7	Δ = 5.4±6.7	Δ = 7.8±4.0	Δ = 4.2±11.0
SVR (dyne·s·cm-5)	1788.7±626.7	1908.2±805.7	1814.1±637.9	1857.5±535.0	1812.8±535.0	1544.0±645.1
PEMI	1748.7±686.1	1694.4±786.5	1710.2±695.0	1734.6±428.5	1733.0±495.3	1562.3±545.2
CER	Δ = 40.0±227.3	Δ = 213.8±205.5	Δ = 103.9±166.9	Δ = 158.8±369.1	Δ = 79.7±207.5	Δ = −18.2±283.4
VFR (ml·s-1)	167.9±79.2	131.2±64.2	107.0±51.2	115.2±33.5	138.4±36.2	159.9±24.4
PEMI	164.4±79.3	125.5±44.3	111.6±32.3	112.9±22.9	127.5±35.4	147.7±22.2
CER	Δ = 3.4±29.4	Δ = 5.7±32.2	Δ = 4.5±22.3	Δ = 2.2±22.1	Δ = 10.4±18.4	Δ = 12.2±24.4

*Parameters response (Δ) are also expressed. HR, heart rate; SV, stroke volume; CO, cardiac output; PEP/VET, pre-ejection period/ventricular ejection time ratio; MBP, mean blood pressure; SVR, systemic vascular resistance; VFR, ventricular filling rate. Values are mean ± SD. See figures for results of statistics*.

**Figure 1:**
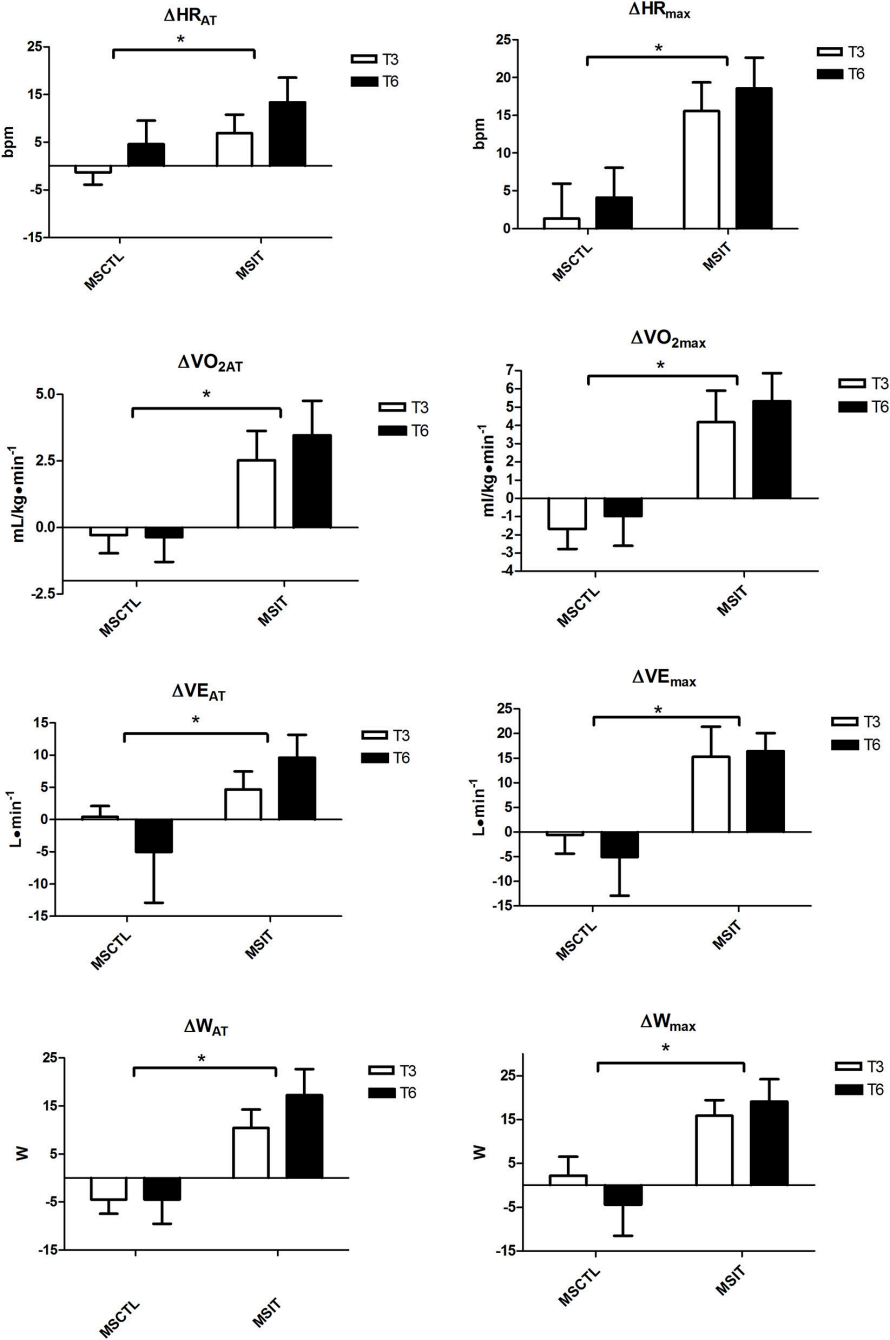
**Variation with respect to T0 in CPT parameters collected at T3 and T6**. Brackets denote a significant group effect, ^*^p < 0.05 between groups. Values are mean ± *SD*.

Figure 1 depicts the variation with respect to T0 in CPT parameters collected at T3 and T6. All variables were significantly affected by group assignment, as in the MSIT group all variables gathered at anaerobic threshold and at maximum workload showed a significant main effect due to group.

**Figure 2:**
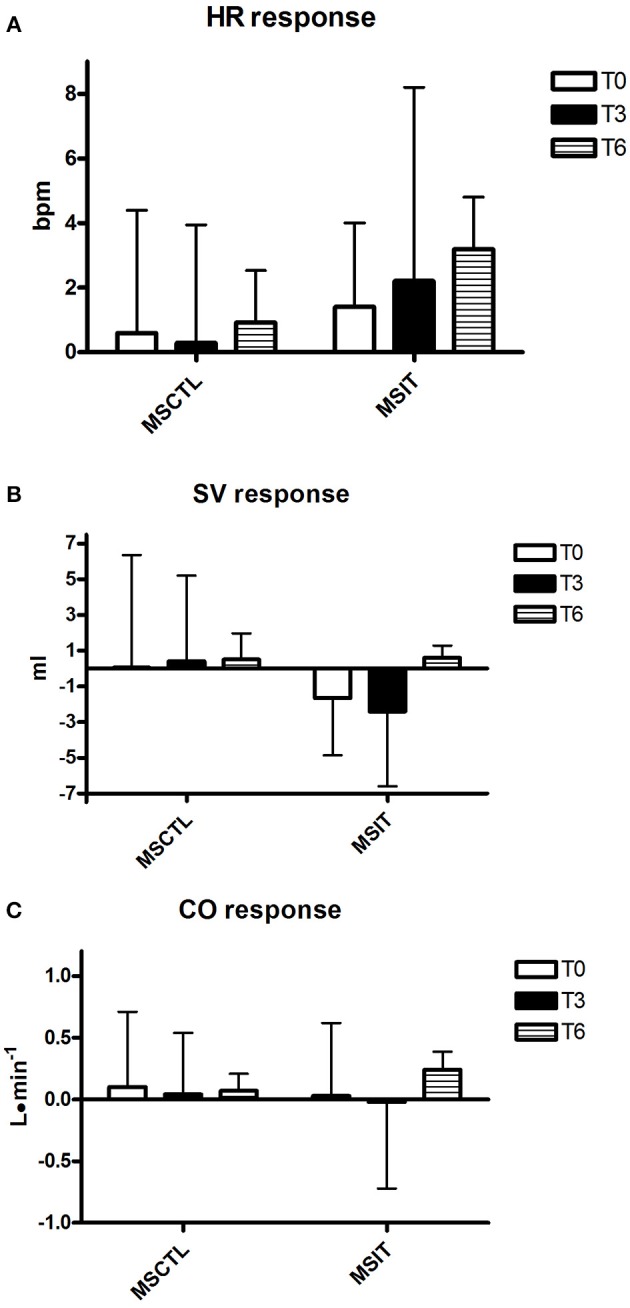
**Responses due to metaboreflex activation in HR (A)**, SV **(B)**, and CO **(C)** at T0, T3, and T6, respectively. Groups are the MSCTL (*n* = 10) and the MSIT (*n* = 11). Values are mean ± SD.

**Figure 3:**
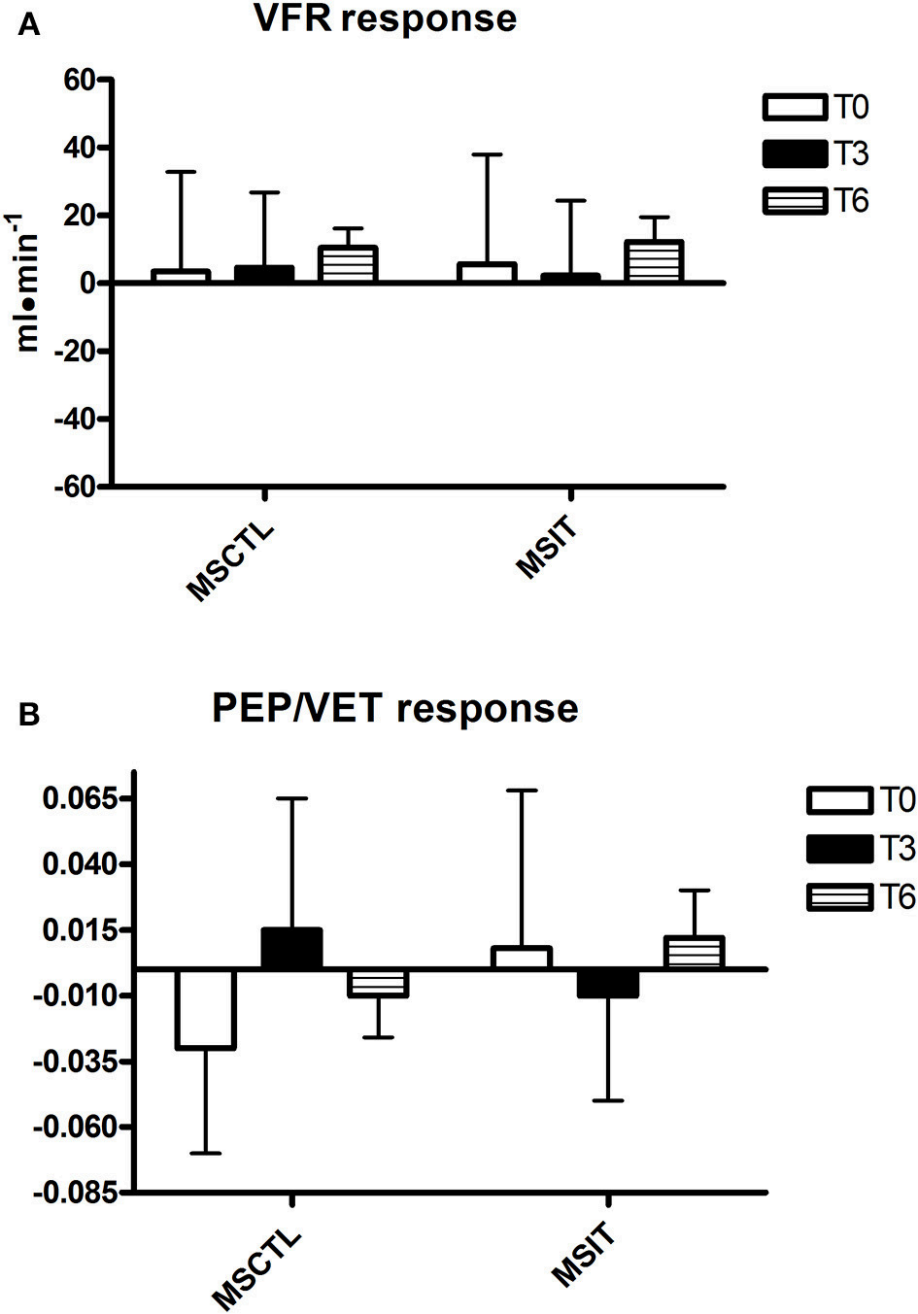
**Responses due to metaboreflex activation in VFR (A)** and PEP/VET **(B)** at T0, T3, and T6, respectively. Groups are the MSCTL (*n* = 10) and the MSIT (*n* = 11). Values are mean ± SD.

**Figure 4:**
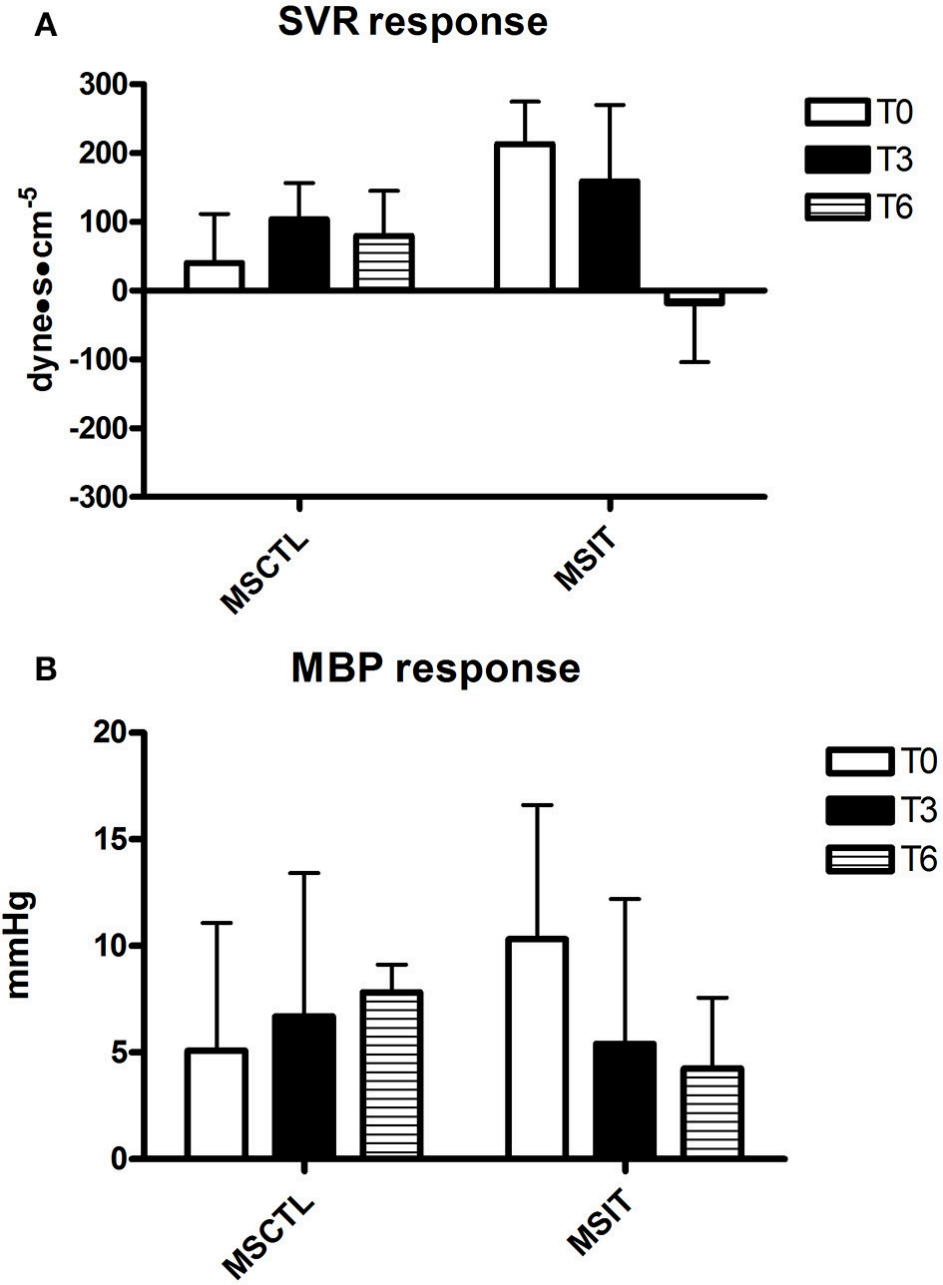
**Responses due to metaboreflex activation in MBP (A)** and SVR **(B)** at T0, T3, and T6, respectively. Groups are the MSCTL (*n* = 10) and the MSIT (*n* = 11). Values are mean ± SD.

Figures 2–4 report hemodynamic responses obtained during the periods of metaboreflex activation, i.e., the difference between the variable's levels at the third minute of recovery from the PEMI test minus the level at the third minute of recovery from the CER test. None of the parameters were affected by group or time at T0, T3, and T6. Notably, the SVR response was always positive (i.e., vascular resistance increased during the PEMI in comparison to the CER test) with the exception of the MSIT group at T6, when SVR actually decreased. However, statistical significance was not reached.

## Discussion

This investigation aimed to ascertain whether the hemodynamic response to metaboreflex activation was positively changed by a period of adapted physical activity. Our reasoning was that physical deconditioning, along with its effect on physical capacity, could counteract the vasoconstriction, and the increase in SVR response shown by MS patients during the metaboreflex. In a recent investigation conducted in our laboratory to discover whether MS patients had a normal hemodynamic response to the metaboreflex, it was found that these patients exaggeratedly increased SVR. It was speculated that this phenomenon was the consequence of the inactive lifestyle of these patients because of their disease (Marongiu et al., 2015). Thus, an intervention able to increase physical capacity may hypothetically counteract this tendency to excessively vasoconstrict the arteriolar bed.

However, results of the present investigation contradict this hypothesis. Indeed, notwithstanding MSIT group patients successfully increased their parameters related to physical capacity as compared to the MSCTL group, this phenomenon was not paralleled by any variation in the hemodynamic response to the metaboreflex. Actually, SVR was unaffected by the training program and no significant variations were detected in neither SV nor CO response in both groups in study.

Our data are in accordance with the concept that a physical intervention is able to improve exercise capacity in MS patients (Ponichtera-Mulcare, 1993; Petajan and White, 1999; Rampello et al., 2007; Dalgas et al., 2008; Andreasen et al., 2011). In reality, the MSIT group substantially increased all CPT parameters related to physical capacity after the period of training in comparison with the MSCTL group, as testified by HR, W, VO_2_, and VE gathered at both AT and maximum workload.

No significant variation in the Expanded Disability Status scale score, neither in the MSCTL nor in the MSIT group was observed. Therefore, the significant improvement in exercise capacity found in the present study was very likely the result of the physical intervention and the reduced sedentary lifestyle. This occurrence confirms the hypothesis that an active lifestyle is a useful behavior to counteract the negative consequences of MS in terms of physical capacity, fatigue perception and physical fitness (Dalgas et al., 2008; Andreasen et al., 2011). Moreover, this finding supports the concept that a sedentary lifestyle is harmful in MS and that sedentary habits worsen the outcome in terms of physical capacity and of the possibility to perform daily-life activities.

Despite the fact that the physical intervention positively affected the physical capacity of patients of the MSIT group, it was unable to significantly change their hemodynamic response during the metaboreflex in comparison with the MSCTL group. Actually, the hemodynamic adjustment during the metaboreflex remained similar between the two groups in study and no significant difference was detected between them, although it should be pointed out that there was a wide inter-subject variability, as testified by the large parameters *SD*. A study with a larger sample size would be useful to ascertain whether reducing variability would result in a different outcome. Furthermore, the physical intervention could not reverse the tendency to increase SVR to reach the target blood pressure during the metaboreflex, with the exception of the MSIT at T6, when the response in this parameter was on average negative. The SVR response found in the present investigation was very similar to that reported in a recent study conducted in our Laboratory in 43 patients with MS (Marongiu et al., 2015). In detail, it was reported that patients with MS on average increased SVR by 137.6 ± 300.5 dyne·s^−1·^cm^−5^ whereas the mean response of healthy controls was −14.3 ± 240.6 dyne·s^−1·^cm^−5^, thereby indicating the absence of vasoconstriction in healthiness. It should however be noticed that, notwithstanding the study by Marongiu et al. (2015) was conducted in a numerous sample (43 patients), a large SD was detected, which suggests that there is a wide inter-subject variability in the SVR response in MS patients.

The fact that the cardiovascular response during the metaboreflex activation was not improved notwithstanding the enhanced exercise capacity suggests that the hemodynamics during the metaboreflex was not related to exertional fatigue in MS patients. It has been hypothesized that the discharges of afferent group III and IV nerve endings in the muscle, which are responsible for the metaboreflex eliciting, are at least in part also responsible for the sensation of fatigue. Feedback from these nervous afferents potentially inhibits central motor drive and facilitates central fatigue, thereby limiting exercise performance (Amann et al., 2008; Mulliri et al., 2016). Thus, fatigue may be hypothetically related to the level of metaboreflex activation. Since in the present investigation we did not find any different hemodynamics before and after the period of training, it is conceivable that the level of metaboreflex activity was similar in these two conditions. However, exercise capacity was higher after training, thereby indicating that probably deconditioning is more important than cardiovascular dys-regulation in the genesis of fatigue in MS patients.

The hemodynamic scenario depicted in the present study resembled the typical response observed in several diseases such as heart failure, obesity, metabolic syndrome, and hypertension, where an excessive sympathetic tone has often been reported (Crisafulli et al., 2007; Delaney et al., 2010; Milia et al., 2015b), whereas in normal subjects vasoconstriction is limited and the target blood pressure is reached mainly by a cardiac output-mediated mechanism (Crisafulli et al., 2003).

It was hypothesized that alterations in skeletal muscle morphology, metabolism, blood flow, and function due to physical deconditioning are important determinants in the altered hemodynamic response to exercise and metaboreflex activation in patients with heart failure. This is known as “the muscle hypothesis” of heart failure (Piepoli and Crisafulli, 2014). Of note, in these patients it appears that physical training can partially reverse this condition (Piepoli et al., 2006), since improving exercise capacity is accompanied by a gradual reduction in metaboreflex-induced vasoconstriction (Piepoli and Crisafulli, 2014). One possible mechanism is that exercise can improve muscle aerobic metabolism, thereby reducing anaerobic end-products accumulation, which are responsible for metaboreflex activation. Moreover, training can counteract the effect of sedentary lifestyle, which induces a shift from type I to type II in muscle fibers composition. It is well known that type II fibers produce a larger amount of metabolic by-products than type I, and in turn this fact can cause an increase in metaboreflex activity with altered hemodynamic response (Piepoli et al., 2008). It is then conceivable that our cohorts of MS patients showed an altered hemodynamic response to the metaboreflex because of a chronic muscle disuse. Actually, it has been reported a shift in fiber type composition from type I fibers to a greater proportion of type II fibers in MS patients as a consequence of deconditioning (Dalgas et al., 2008).

It is however a matter of fact that in the present study there was the lack of any significant improvement in the hemodynamic response to the metaboreflex, although future research with more patients is warranted. It should be kept in mind that MS is a demyelinating disease, where the progression of nervous fibers degeneration is unpredictable and does not depend on patients' physical capacity. Hence, it is possible that the lack of improvement in the hemodynamic response during the metaboreflex could simply depend on irreversible damages at the level of central nervous system. For instance, lesions at the level of the spinal cord, especially at the dorsal horn, where there are important sensory inputs for the metaboreflex, could account for the altered hemodynamics that these patients showed after the period of training.

### Limitations of the study

One limitation of the present study is the reduced number of patients enrolled that could potentially have masked the effect of the training intervention on the cardiovascular regulation. While the sample size did suffice to discover positive effects on CPT parameters due to training, it was probably not large enough to identify effects on hemodynamic variables in response to the metaboreflex. Thus, it is possible to speculate that the lack of significant reduction in the SVR response was the result of the small sample size of the present investigation. It can be hypothesized that increasing the number of subjects enrolled would have resulted in a significant reduction in the SVR response. Further research is warranted on this topic to better clarify this point. Another limitation is that it is possible that the period of intervention, i.e., 6 months, was too short to successfully affect the cardiovascular regulation. It can be speculated that a longer period of intervention (i.e., 12 months) would have led to a more pronounced reduction in SVR response. In a similar experimental setting conducted in heart transplant recipients, where a 12-month period of observation was applied after transplant, the maximum reduction in SVR response was obtained after 12 months (Crisafulli et al., 2013). In the quoted study, it was concluded that, despite improved cardiac function, the impairment in the cardiovascular regulation in response to muscle metaboreflex after heart transplant was evident only after several months. That is, it takes months to re-set cardiovascular regulation after a long period of sedentary habits. Since our MS patients had sedentary habits, it can be speculated that they needed a longer period of training to completely reverse the consequence of sedentary a lifestyle due to their pathology. A final limitation is that we did not take into consideration parameters related to quality of life in the MS patients. Instead, only variables related to exercise capacity and cardiovascular regulation were considered. Therefore, the present data do not allow to discover whether or not exercise capacity and/or cardiovascular regulation contribute to quality of life in these patients.

In conclusion, results of the present study demonstrate that a 6-month period of adapted physical training improved physical capacity and cardiopulmonary indexes related to it in patients suffering from multiple sclerosis. However, it was unable to counteract the tendency of these patients to vasoconstrict the arteriolar bed to achieve the target blood pressure in response to the muscle metaboreflex. Increasing the sample size and the period of intervention is mandatory to better understand whether a more positive outcome could be achieved by physical training. Further research is needed to confirm this hypothesis.

## Author contributions

SM, SO, EC, EM, AC conceived the study, conducted experiments, designed and wrote the paper. MP, GP, FT, MGM, and AC conceived the study, designed and wrote the paper. AD, MM, AL, FeC, FrC, and GC conducted experiments, designed and wrote the paper. All authors read and approved the final version of the manuscript.

## Funding

This study was supported by the University of Cagliari, the Regione Sardegna (legge regionale n°7 Agosto 2007, annualità 2011), and the Italian Ministry of Scientific Research.

### Conflict of interest statement

The authors declare that the research was conducted in the absence of any commercial or financial relationships that could be construed as a potential conflict of interest.
